# What’s Wrong With Factory Farming?

**DOI:** 10.1093/phe/phu001

**Published:** 2014-02-07

**Authors:** Jonathan Anomaly

**Affiliations:** Duke University

## Abstract

Factory farming continues to grow around the world as a low-cost way of producing animal products for human consumption. However, many of the practices associated with intensive animal farming have been criticized by public health professionals and animal welfare advocates. The aim of this essay is to raise three independent moral concerns with factory farming, and to explain why the practices associated with factory farming flourish despite the cruelty inflicted on animals and the public health risks imposed on people. I conclude that the costs of factory farming as it is currently practiced far outweigh the benefits, and offer a few suggestions for how to improve the situation for animals and people.

Factory farming involves raising livestock in densely populated environments often called ‘concentrated animal feeding operations’. ^1^ Common practices include packing pregnant pigs into gestation crates so small they cannot turn around, placing egg-laying hens in cages stacked on top of one another in massive enclosed buildings and raising cows on feedlots rather than the grass pastures many of us associate with ruminants. ^2^ Because of the stress induced by these conditions, including the constant frustration of their natural instincts, many animals develop compromised immune systems, and without a steady course of antibiotics, many more would become sick and die of bacterial infections. Thus, antibiotics are often used to compensate for conditions that would otherwise make it impossible to raise animals ( Alliance for the Prudent Use of Antimicrobials, 2010 ).

The practices that comprise factory farming evolved as a result of competition between firms to produce commodities—mainly milk and meat—at minimal cost. Competition usually benefits consumers. Factory farming has lowered the price of animal protein, and this is a real boon for poor and middle-class consumers. But there are at least three moral problems with factory farming, and none of them is factored into the price of the animal products they create. These include the spread of pathogenic viruses, the diffusion of antibiotic-resistant bacteria into our shared microbial environment and the immense cruelty suffered by animals in confined conditions.

## Animals and Influenza

Experts agree that most (and perhaps all) strains of the influenza virus that infect human beings originated from contact with other animals, especially domesticated birds and pigs in Asia ( Crawford, 2000 : 95). The advent of animal agriculture brought a steady supply of protein to people, but it also increased the transmission of viruses carried by animals, and spurred the evolution of existing viruses.

There are several reasons factory farms seem to elevate the risk of novel viral outbreaks—especially variations of avian and swine flu. First, crowding animals together in close confinement can induce stress and suppress their immune systems, raising parasite loads and making animals more susceptible to infections; second, as all of us have learned after catching a cold in school or at work, viral transmission is facilitated by animals being kept in proximity to one another; third, close contact between different species of animals gives viruses a continuous opportunity to mutate and reassort to create new strains; fourth, many factory farms confine animals to indoor spaces that lack adequate sunlight or ventilation, which allows viruses to survive longer without a host; and finally, because animals on factory farms are often genetically similar, they can be more susceptible to specific parasites ( Crawford, 2000 ; Greger, 2007 ).

The situation on factory farms is in some ways analogous to that of overcrowded prisons ( Schmidt, 2009 ). Infectious diseases flourish in prisons for some of the same reasons: high stress and poor nutrition can impair people’s immune systems, and crowding permits a quick transfer of microbes and a continuous supply of hosts. This is one reason many experts believe pathogenic viruses like hepatitis have spread more rapidly in crowded prisons than in the surrounding population ( Bick, 2007 ).

Most people already understand that crowding can spread sickness, and compromised immunity makes people more susceptible to infection, but few people understand how crowding different species together—as occurs on factory farms and in live animal markets—might hasten the evolution of new strains of virus. According to Dorothy Crawford, ‘[b]ird viruses usually lack the receptor binding protein needed to infect human cells, but some domestic animals like pigs and horses are susceptible to both bird and human strains. So gene swapping between human and bird strains often occurs in pigs or horses, causing a major genetic change in the virus make-up called an *antigenic shift* . Occasionally after this mixing a “new” virus strain emerges that can infect and spread in humans, and as the population is completely naïve to this “new” strain it can spark a pandemic’ ( Crawford, 2007 : 205).

Viruses have long jumped between species, but the advent of animal agriculture increased opportunities for viral transmission between animals and humans. The Spanish flu pandemic of 1918, which infected half of the world’s population and killed tens of millions of people, is thought to have arisen in farm animals. Although this particular strain cannot be blamed on practices that began in the late 20th century, our current practices increase the risk that new versions of existing viruses will emerge. It should be emphasized that in most cases, it is impossible to track the exact origin and evolutionary progress of any particular strain of flu. Scientists instead look for patterns of correlation between sites of initial infections, and rely on general knowledge about the conditions that facilitate the emergence and transmission of viruses.

Regardless of the origin of specific outbreaks of swine and avian flu, the general trend seems to implicate factory farming as a significant cause of many new strains: ‘there is no doubt that we are in the midst of the worst ever recorded flu pandemic in birds. The [H5N1] virus started life as a harmless infection in the intestines of wild birds and jumped to domestic chickens in the 1990s, where modern intensive farming techniques gave it the opportunity to adapt and evolve … . And now this virulent strain has not only crossed back into wild fowl but has increased its host range to include other birds … and even some mammals such as cats’ ( Crawford, 2007 : 208).

Although I have focused on different strains of influenza, animals share many other viruses, even if only a small number induce death or disease when they jump species. The morally interesting question is whether we can justify practices that increase the likelihood of new viral epidemics.

It is conceivable that new strains of viruses that arise on factory farms will eventually lose their virulence and strength. When viruses are confined to a specific population, they tend to become weakened as they co-evolve with the animals that host them. This occurs because from the virus’s standpoint—from the standpoint of the ‘selfish’ genes that comprise viruses—a host is better alive than dead, as a live host can create more copies of the virus and spread it to more people.

However, it can take many years for a virus to become benign ( Crawford, 2000 ), and in the intervening period, it can decimate populations. So the fact that in the long run viruses tend to lose their virulence—their ability to cause disease or death in the animals that host them—does not suggest that we should continue to allow factory farmers to house their animals in extreme confinement. ^3^

## Antibiotic Resistance

In recent years, awareness of the problem of antibiotic resistance has grown as bacterial diseases ranging from tuberculosis to gonorrhea have become increasingly costly or difficult—and in some cases, impossible—to treat by existing antibiotics ( United States Centers for Disease Control and Prevention, 2013 ). Many people now understand the basic evolutionary principle that our increasing use (and misuse) of antibiotics fuels the evolution and dispersion of antibiotic-resistant bacteria. But fewer people are aware of the connection between the widespread use of antibiotics in livestock and the emergence of new patterns of antibiotic resistance in people.

Resistance to antibiotics arises in farm animals for the same reason it does in people. For billions of years, bacteria have been competing with each other and with plants, fungi and animals for scarce resources. Although most of these relationships have become mutualistic (beneficial to both parties) or commensal (neither harmful nor beneficial to both parties), some are parasitic relationships in which bacteria benefit at the expense of their host. When bacteria parasitize other organisms, natural selection rewards genetic mutations and immune responses that allow their victims to fight back. The arsenal that organisms have evolved to defend themselves against exploitation includes chemical weapons that destroy bacteria, and enzymes that disrupt DNA synthesis to prevent their replication. Bacteria have responded in kind by developing sophisticated defenses, including membranes that block antibiotic absorption ( Delcour, 2009 ), enzymes that degrade the efficacy of antibiotics ( Wright, 2005 ) and efflux pumps that eject antibiotics that have already been absorbed ( Kumar and Schweizer, 2005 ).

One might think that organisms with an adaptive immune system would eventually find a way to resist bacterial exploitation. To some extent this occurs, which explains the existence of endogenously produced antibiotics in many organisms, and natural immunity to the deleterious effects of some bacteria in others. But bacteria have responded with a creative way of evolving quickly. Horizontal gene transfer through conjugation and transduction allows bacteria to acquire genes from other bacteria, from phage viruses that parasitize them and occasionally from unwilling hosts. This allows bacteria to exploit mutations and gene sequences that arise in other organisms, which is one reason most scientists see no way of developing an antibiotic that permanently removes the threat of harmful bacteria. The challenge instead is to find specific antibiotics that kill harmful bacteria, undermine their virulence or prevent them from replicating long enough for an immune system to clear them from an animal’s body.

Nearly half of all antibiotics worldwide are given to farm animals to promote growth and prevent diseases in the crowded quarters in which livestock are increasingly kept, and in the US, an estimated three quarters of all antibiotics go directly to livestock on factory farms ( US GAO, 2011 ). For many years, public health experts have warned about the dangers of using large quantities of antibiotics in farm animals, especially when they are used at sub-therapeutic doses over long periods, as this creates an ideal environment for bacteria to evolve and spread resistance to antibiotics ( Gorbach, 2001 ). Antibiotics are administered at low levels because they can speed the growth of some animals by increasing nutrient absorption and preventing infections in cramped conditions ( McEwen and Fedorka-Cray, 2002 ). Using antibiotics for non-therapeutic purposes gives farmers a small but significant advantage over those who decline to use them, thus creating a negative sum game in which the rational profit-maximizing choice for each farmer gives no farmer any particular advantage over others, but leaves nearly all animals and people worse off.

Animals are worse off because of the cruel conditions in which they are kept. Farmers are no better off using antibiotics for non-therapeutic purposes if their competitors are also permitted to use them. People are worse off because antibiotic-resistant bacteria often find their way into human hosts.

Antibiotic-resistant bacteria that arise on factory farms can spread to human hosts in a number of ways. First, those who work on farms and handle animals or raw meat can pick up antibiotic-resistant bacteria from animals who have it, and transfer it to other people; second, some bacteria survive in meat even after it is cooked, and are transferred directly to those who eat it; third, animal waste from factory farms that contains antibiotic-resistant bacteria is often used to fertilize crops, and some of these bacteria infect people who either work with crops or consume them; and finally, as bacteria do not respect physical or biological borders, some are transferred to animals and streams around factory farms ( McEwen and Fedorka-Cray, 2002 ; Casey *et al.* , 2013 ).

In a recent overview of antibiotic resistance on US farms, the Environmental Working Group found that among the most common meats bought in US supermarkets, 81% of turkey, 69% of pork chops, 55% of ground beef and 39% of chicken contained antibiotic-resistant bacteria ( Undurraga, 2013 ). By themselves, these numbers should not be alarming, as many bacteria have been carrying antibiotic-resistant genes for millions of years. It is possible for resistant bacteria to spread from animals to humans, or from humans to animals, so the misuse of antibiotics among people may be (at least partly) responsible for recent increases in resistance among bacteria that colonize farm animals ( Singer, 2003 ). However, recent increases suggest that factory farming practices are largely responsible for antibiotic resistance among farm animals, and thus in the meat that derives from them. While experts argue about whether most resistance comes from the misuse of antibiotics (such as their use for growth promotion), or whether it comes simply from the total quantity used, there is clear evidence that more use in people or animals creates more resistance in the bacteria that colonize them (Wegener, 2003a), and that reducing their use in farm animals in countries like Denmark has led to less resistance (Wegener, 2003b).

Although it is necessarily imprecise, we can measure the increased prevalence of antibiotic-resistant bacteria over time. For example, between 2002 and 2011, multidrug-resistant *Salmonella* in raw chicken in the US has increased from ∼20 to 45%, and in turkey during the same period, it increased from ∼20 to 50% ( Undurraga, 2013 ). Human deaths from multidrug-resistant *Escherichia coli* derived from poultry are on the rise, and this is likely to be true for many pathogenic bacteria derived from farm animals ( Collignon *et al.* , 2013 ). Unfortunately, withdrawing antibiotics from animal feed does not work especially quickly. Just as it takes time for bacteria to acquire and spread genes that confer resistance, it often takes time for them to lose these genes when antibiotics are withdrawn ( Lenski, 1998 ). This is because although genes that confer resistance are costly to carry, the costs are often minimal and some genes can encode for the conditional expression of resistance, so that resistance genes are only phenotypically expressed in bacteria when antibiotics are present ( Andersson and Levin, 1999 ). And although the prevalence of resistance genes typically falls over time when antibiotics are withdrawn, it takes a long time to approach 0. So when antibiotic use is resumed, even a small number of bacteria with antibiotic-resistant genes can spread rapidly within and between different bacterial populations ( Salyers and Amabile-Cuevas, 1997 ). This suggests that it may take considerable time before removing antibiotics in agriculture restores their efficacy.

While most European countries have phased out the sub-therapeutic use of antibiotics in livestock over the past decade, the US and other countries have been slow to respond—presumably because there are significant upfront costs to changing the way farm animals are fed and housed, and because farmers with lobbying power fear losing market share to less scrupulous farmers in other countries who continue to use factory farming techniques that necessitate antibiotics.

## Animal Cruelty and Public Policy

Philosophers argue about whether animals have rights, and if so where these rights come from. These are important arguments to have, but any plausible theory will hold that sentient creatures capable of feeling pain and frustration have interests that deserve protection. The problem is that the interests of animals and people can come into conflict. People have an interest in advancing medical research and consuming cheap protein, and animals have an interest in being able to exercise their instincts, or at least being free from gratuitous pain and frustration. When interests collide, the differences between various theories of animal rights will come to the fore. However, we can start with the assumption that any theory of animal welfare worth taking seriously will include a *pro tanto* obligation not to inflict cruelty on animals without sufficient justification.

Some think that rationality or consciousness is a necessary condition for moral standing. ^4^ Others suggest that sentience is sufficient for moral standing, so that all sentient animals deserve to have their interests protected. The claim that animals are equal in the sense of having their interests equally considered does not imply that they should be treated the same. Instead, the idea is that having an interest means that moral agents should take these interests into account when deciding what to do. The fact that a pig has interests does not imply that it should be given the right to own a home or drive a car, but rather that we should minimize unnecessary pain and frustration ( Singer, 1976 ), perhaps by according it legal rights, and by requiring farmers to abide by certain animal welfare standards.

Another view is that although a variety of qualities like rationality, sentience and empathy give animals moral standing, there is no precise combination of qualities that clearly separates animals with and without rights. Call this view pluralism. James Rachels seems to endorse this view:

There is no characteristic, or reasonably small set of characteristics, that sets some creatures apart from others as meriting respectful treatment. That is the wrong way to think about the relation between an individual’s characteristics and how he or she may be treated. Instead we have an array of characteristics and an array of treatments, with each characteristic relevant to justifying some types of treatment but not others. If an individual possesses a particular characteristic (such as the ability to feel pain), then we may have a duty to treat it in a certain way (not to torture it), even if that same individual does not possess other characteristics (such as autonomy) that would mandate other sorts of treatment (refraining from coercion). [ Rachels, 2004 : 169].

Some find this view unsatisfying because it fails to draw clear lines or to list off a single set of obligations that we owe to all creatures with moral standing. But the fact that our moral universe is more complicated than we would like it to be does not imply that pluralism is false. Regardless of their differences, pluralists like Rachels and consequentialists like Singer agree that farm animals should be guaranteed minimally decent treatment, and that using them as mere means to our ends is wrong.

Animals used for food are treated differently around the world, and even Western countries offer different protections. For example, while the European Union (EU) has robust anti-cruelty laws that apply to all member states, the US federal government affords no protection at all to animals raised for food. The US Animal Welfare Act passed in 1966 exempts most animals that humans come into contact with from protection against cruelty:

Farm animals, such as domestic cattle, horses, sheep, swine and goats that are used for traditional, production agricultural purposes are *exempt from coverage by the AWA* [emphasis added]. Traditional production agricultural purposes include use as food and fiber, for improvement of animal nutrition, breeding, management or production efficiency, or for improvement of the quality of food or fiber. ^5^

The federal government has delegated this responsibility to state governments, and many states have created animal welfare laws designed primarily to protect the interests of meat producers, and companion animals like dogs and cats, while excluding farm animals of similar or greater sentience from similar protection. Most states make it difficult to prosecute violations of animal welfare laws, and have relatively weak anti-cruelty provisions, which count a practice as unacceptably cruel only if it violates existing practices.

According to Wolfson and Sullivan,

In a rapidly growing trend, as farming practices have become more and more industrialized and possibly less and less acceptable to the average person, the farmed-animal industry has persuaded the majority of state legislatures to actually amend their criminal anticruelty statutes to simply exempt all ‘accepted’, ‘customary’ or ‘normal’ farming practices ( Wolfson and Sullivan, 2004 : 212).

These provisions would be considered outrageous if applied to humans. Imagine a world in which some humans are considered the property of others, and the question is how the property owners should be allowed to treat their subjects. Some owners argue that it would be costly to improve the already awful standards, so we should only regard acts as cruel if they violate practices that already exist. While there are clear differences between human and non-human animals, defining morally acceptable practices by reference to whatever is currently done is morally perverse, and it precludes virtually any improvement in existing standards.

In recent years, some states have extended more protection to farm animals. For example, in 2008, California voters passed the Prevention of Farm Animal Cruelty Act, which requires that ‘calves raised for veal, egg-laying hens and pregnant pigs be confined only in ways that allow these animals to lie down, stand up, fully extend their limbs and turn around freely.’ ^6^ While this is a slight step forward, it may also induce farmers to move to other states to continue their cruel but cost-saving practices. States like Nevada have made significant efforts to lure farmers out of California. ^7^

In contrast to the US (and much of the rest of the world), the EU has enacted strong protections for farm animals, and some individual states have passed laws that exceed these standards. For example, while the entire EU has banned the use of gestation crates for pigs and battery cages for hens, Germany has banned cages and crates for all farm animals. In Germany, farmers are required to raise hens in large barnlike aviaries, to allow other animals to move around with some degree of freedom, and to have straw or grass bedding, rather than sleeping on concrete floors surrounded by metal cages ( Wolfson and Sullivan, 2004 ).

An advantage of the EU’s approach to animal welfare is that it establishes minimally acceptable requirements that states and farmers are free to exceed. Therefore, it reduces the collective action problem in which farmers who would prefer to provide an enriched environment for animals fear that other farmers will exploit this concern by using cheaper techniques that externalize the moral costs of production.

It might be argued that if people are concerned about the treatment of animals, or the threat of zoonotic epidemics and antibiotic resistance, they should change their consumption rather than using the power of the state to force producers to alter their production practices. While I agree that people who understand the costs of factory farming have a moral obligation to change the way they shop for meat (for example, to look for labels like ‘certified humane’ and ‘free range’), and that some people who do not understand the moral issues surrounding factory farming are culpably ignorant and have an obligation to familiarize themselves with the issues, I do not think we should simply assume that consumers will voluntarily change their habits.

First, some ignorance of morally repugnant practices is, in the economic sense, rational. Because we have limited time, and information is costly to gather and process, consumers are often rationally ignorant about how their actions and consumption choices affect other people and animals. It is difficult, and arguably undesirable from a social standpoint, to expect consumers to know everything about how the products they consume are made. In fact, this is the point of prices in a well-functioning market: consumers and producers do not need to understand how everything is made to act in ways that tend to make others better off ( Hayek, 1945 ). But this is only true when prices capture most of the costs and benefits generated in producing pencils and paperclips, and other consumer goods. When milk and meat are produced in such a way that the costs to people and animals are not factored into the price of production, we are not necessarily better off, and our ignorance can lead us to make choices that we would not make if we were aware of the harm they impose on others.

Second, the core function of a liberal government is to produce public goods and prevent people from imposing unwarranted harms on each other. Giving people the discretion to consume factory-farmed foods allows them to inflict cruelty on animals, and to inflict significant health costs—even death—on other people. While the harm to animals is direct, the harm to other people is probabilistic and diffuse. Each person’s consumption of meat from factory-farmed animals merely contributes to a process that significantly elevates the risk of harm to other people in the form of antibiotic-resistant infections, or new viral infections that arise in birds and pigs.

Since some people will continue to consume factory-farmed products because it is cheaper than the alternatives, or because they are ignorant of the harms associated with these products, we cannot rely solely on social norms and moral outrage to drive farmers to alter their practices, nor can we rely on farmers to voluntarily phase out factory farming, as most farmers who act this way will be driven out of business by less altruistic competitors. ^8^ Instead, governments should require factory farmers to change the way they raise animals.

An obvious starting point is for the rest of the world to follow the EU in banning the use of battery cages for hens (which typically involve stuffing half a dozen hens into cages so crowded they can barely move) and gestation crates for veal and sows. By requiring farmers to use straw or other bedding for animals and increase roaming space and access to fresh air, we can marginally increase their comfort and decrease the stress that leads to compromised immunity. This alone would significantly increase welfare and reduce the risk of zoonotic viral infections. It would also reduce the need to administer antibiotics to prevent infections brought on by crowding.

The US should also follow Europe in banning the sub-therapeutic use of antibiotics to promote growth in farm animals ( Lessing, 2010 ), and should tax the use of antibiotics for therapeutic purposes, using the revenue to fund research into new vaccines and new antibiotics ( Vagsholm and Hojgard, 2010 ; Anomaly, 2013 ). One study suggests that when we tax pollution—in this case, the use of antibiotics that leads to antibiotic resistance—and use the revenue generated from the tax to address the source of pollution or compensate victims, public support for the tax increases ( Kallbekken *et al.* , 2011 ). It is also arguably more efficient and fair to tax practices that produce social costs rather than activities that are socially beneficial ( Anomaly, 2010 ).

At the very least, the US and other countries should prohibit the use of all medically important antibiotics when they are used simply for the purposes of growth promotion, or as a way of compensating for crowded and unhealthy conditions on factory farms. This is what the US Preservation of Antibiotics for Medical Treatment Act proposes, although even if it eventually passes, there is some worry that it may not go far enough because sometimes resistance to non-medically important antibiotics can also confer resistance to medically important ones. ^9^ Because we share a microbial environment, the overuse of antibiotics aimed at particular bacteria can increase the prevalence of antibiotic resistance among other bacteria that are likely to affect human health. Thus, instead of allowing farmers to decide on the kind and quantity of antibiotics to give to their animals, we might at least require veterinary oversight. Allowing farmers to administer antibiotics indiscriminately is tantamount to allowing them to decide how much harm they would like to inflict on other people.

One potential problem with banning antibiotics for growth promotion, and requiring veterinary supervision and prescription for administering antibiotics to sick animals (or as prophylaxis for potentially sick animals), is that farmers might pressure veterinarians to prescribe antibiotics when they are not really needed. More plausibly, in the absence of other requirements like increased roaming space, farmers might actually need antibiotics for sick animals—not because animals naturally get sick a lot, but because the conditions on factory farms ensure that animals will be infected with pathogenic bacteria. ^10^ This suggests the need to impose a complementary package of requirements on farmers that both improves animal welfare and decreases the transmission of disease.

It is impossible to say with precision what the total cost of imposing new requirements on farmers would be. If the cost was large, this could be a real loss for people with less income. But the argument from cost is not decisive.

Evidence from Europe indicates that the cost of complying with more stringent rules may not be as high as farmers anticipate. For example, in Denmark, the extra cost so far of implementing standards that increase animal welfare and decrease antibiotic use is estimated at $1 per pig ( Wegener, 2003b : 448). It is likely that forcing farmers in the US and China to switch from intensive methods would impose greater costs, as both countries currently use much more confinement and antibiotics than Denmark ever did. The problem with estimating the cost of changing methods is that organizations representing animal welfare advocates and factory farmers give different estimates, and it is too early to know precisely how new provisions in Europe and California will impact prices, as they are just beginning to come into effect.

Another reason to think the argument from cost is not decisive is that although meat has been a cheap and sometimes necessary source of high-value protein for humans throughout much of our history, a nutritionally adequate diet does not require the consumption of meat, and certainly does not require the amount of meat consumed by people living in Japan or the US ( Smil, 2013 ).

Finally, when the relative price of meat increases, markets will reward research into synthetically created meat, derived from stem cells, which may eventually be healthier and cheaper than ‘naturally’ created meat. ^11^

The argument that phasing out factory farming would unfairly harm the poor by increasing the cost of meat is not a sufficient reason for failing to act. Many poor people around the world would still be able to consume humanely raised animal products, and people in destitute poverty may have to turn to grains and legumes for most of their protein (as they already do). But being forced by circumstance to consume less meat than one would like does not give people the right to consume or produce food in a way that inflicts unwarranted harm on other people or animals.

## Notes

1. According to the EPA, a Concentrated Animal Feeding Operation (CAFO) is a farm in which ‘animals are kept and raised in confined situations. CAFOs congregate animals, feed, manure and urine, dead animals and production operations on a small land area. Feed is brought to the animals rather than the animals grazing or otherwise seeking feed in pastures, fields or on rangeland.’ http://cfpub.epa.gov/npdes/home.cfm?program_id=7 [accessed 10 October 2013].

2. For a more thorough description of conditions on factory farms, see Singer and Mason, *The Way We Eat* ( 2006 ), the HBO documentary, *Death on a Factory Farm* (2009), and Wolfson and Sullivan, ‘Foxes in the Henhouse’ ( 2004 ).

3. My point here is more about the degree of animal confinement, or stocking density, rather than the size of the farming operation. It may be that larger animal farms that enclose the animals from contact with wildlife are safer, and make disease surveillance more cost-effective, than backyard chicken or pig farms.

4. Immanuel Kant is generally thought to have held this view, although he did acknowledge indirect duties toward non-rational animals.

5. http://www.aphis.usda.gov/animal_welfare/policy.php?policy=17 [accessed 10 January 2014].

6. http://ballotpedia.org/wiki/index.php/California_Proposition_2,_Standards_for_Confining_Farm_Animals_%282008%29 [accessed 10 January 2014].

7. http://online.wsj.com/news/articles/SB126334191947626965 . Wall Street Journal [accessed 26 November 2013].

8. The biggest flaw in current US policy is that it sets recommendations rather than binding requirements for farmers to limit their use of antibiotics, and to alter their practices to increase animal health ( United States Government Accountability Office, 2011 ).

9. http://www.govtrack.us/congress/bills/113/hr1150 [accessed 10 January 2014].

10. There is some evidence that in Denmark, where non-therapeutic antibiotics were banned in 2000, farmers have steadily increased the use of antibiotics for therapeutic purposes, although net use is still down significantly ( United States Government Accountability Office, 2011 : 40). This problem could be minimized by requiring veterinary prescription, and by imposing modest taxes or fees on the use of antimicrobials. If the taxes were too high, this might lead to an increase in easily preventable animal suffering. But even a modest tax could deter superfluous use, force farmers to keep animals in better conditions and raise revenue for research into alternatives to existing practices, including the development of new kinds or classes of antibiotics.

11. The latest version of synthetic meat is being bankrolled by Google co-founder Sergey Brin: http://www.dailymail.co.uk/sciencetech/article-2384715/At-tastes-meat–Worlds-test-tube-artificial-beef-Googleburger-gets-GOOD-review-eaten-time.html [accessed 21 September 2013].
